# Wearable Bioimpedance Monitoring: Viewpoint for Application in Chronic Conditions

**DOI:** 10.2196/22911

**Published:** 2021-05-11

**Authors:** Willemijn Groenendaal, Seulki Lee, Chris van Hoof

**Affiliations:** 1 Imec the Netherlands / Holst Centre Eindhoven Netherlands; 2 Imec Leuven Belgium; 3 One Planet Research Center Wageningen Netherlands; 4 Department of Engineering Science KU Leuven Leuven Belgium

**Keywords:** wearable monitoring, bioimpedance, impedance pneumography, impedance cardiography, body composition, imaging

## Abstract

Currently, nearly 6 in 10 US adults are suffering from at least one chronic condition. Wearable technology could help in controlling the health care costs by remote monitoring and early detection of disease worsening. However, in recent years, there have been disappointments in wearable technology with respect to reliability, lack of feedback, or lack of user comfort. One of the promising sensor techniques for wearable monitoring of chronic disease is bioimpedance, which is a noninvasive, versatile sensing method that can be applied in different ways to extract a wide range of health care parameters. Due to the changes in impedance caused by either breathing or blood flow, time-varying signals such as respiration and cardiac output can be obtained with bioimpedance. A second application area is related to body composition and fluid status (eg, pulmonary congestion monitoring in patients with heart failure). Finally, bioimpedance can be used for continuous and real-time imaging (eg, during mechanical ventilation). In this viewpoint, we evaluate the use of wearable bioimpedance monitoring for application in chronic conditions, focusing on the current status, recent improvements, and challenges that still need to be tackled.

## Introduction

Chronic diseases are currently a major challenge for the global health system [1]. Worldwide, over 70% of deaths are attributed to noncommunicable diseases (NCDs) and mental health. In addition, NCDs are a leading cause of morbidity and disability, including cardiovascular disease, chronic respiratory diseases, cancer, and diabetes [2]. Specifically in the United States, approximately half of the overall population is suffering from one or more chronic diseases [3]; 6 in 10 adults have at least one chronic disease and 4 in 10 adults are suffering from two or more chronic diseases [4]. This not only poses a huge burden on the health care system but is also an economic burden, as chronic diseases account for 86% of the total health care costs in the United States [5].

Some of the main aspects attributing to these high health care costs are the emergency room visits and hospitalizations resulting from acute exacerbations in chronic diseases [6]. At present, these diseases are typically managed based on a few office visits per year [7]. Several studies have shown that more frequent monitoring could lead to early detection of exacerbations such as in heart failure [8] and in asthma [6]. This indicates that continuous or frequent monitoring could also play a role in the management of the large number of patients suffering from chronic diseases [9].

Wearable sensor technology, possibly combined with artificial intelligence (AI), is one of the techniques that provides this type of monitoring. Consequently, the wearable technology market has increased rapidly in recent years. Different wearables have been developed, ranging from simple medical alarms (St John) that people can press when needing help to vital sign patches for monitoring electrocardiogram (ECG) signals (ePatch BioTelemetry Inc and Vista Solution VitalConnect) and cuff-based blood pressure measurements in a watch (HeartGuide Omron Healthcare Inc). At the same time, different AI methods have been developed and applied to physiological data, ranging from supervised techniques for automatic detection of sleep apnea from the ECG [10] to unsupervised heart rate detection with liquid states [11].

A promising sensing method is wearable bioimpedance monitoring. In this paper, we define a wearable bioimpedance monitoring system as an electronic device containing a bioimpedance sensor capturing the bioimpedance of the wearer that is worn close to or on the surface of the skin, and that allows the wearer to move freely during daily living conditions (ie, that is not attached to any main power supply or desktop device). Bioimpedance is a versatile sensing technology that can be used for a wide array of clinical and lifestyle applications, ranging from body fluid monitoring [12] to gesture monitoring [13] and to monitoring of hemodynamic parameters [14]. In addition, bioimpedance is a noninvasive technology and is of relatively low cost. Specifically, in chronic disease management, bioimpedance has, for example, been explored to monitor patients with asthma [15], heart failure [8], and end-stage kidney disease (ESKD) [16]. Table 1 lists some of the commercially available devices and their application areas as of November 2020. There are still several challenges for the full integration of wearable bioimpedance monitoring into the clinical health care system. Some of these challenges are specific to bioimpedance; however, many are general to wearable monitoring. These challenges include data reliability [17,18], patient usage and compliance [19,20], integration into electrical health records [21,22], actionable insights provided to the user, and the still limited number of clinical trials demonstrating a medical benefit [23]. Here, we discuss the versatile application areas for wearable bioimpedance monitoring, along with the current status, remaining challenges, and future outlook.

**Table 1:** Wearable bioimpedance devices currently available on the market.

Product	Company	Technology	Application	Market
Auraband	Aura devices, Wilmington DE, USA	Wrist band, hand-to-hand BIVA^a^	Body composition	Consumer
Inbodyband	Inbody, Seoul, Korea	Wrist band, hand-to-hand BIA^b^	Body composition	Consumer
CoVa Monitoring system	ToSense (acquired by Baxter International)	Necklace, thoracic bioimpedance	Heart failure	Medical
Shimmer3 Ebio unit	Shimmer, Dublin, Ireland	Module attached with chest strap, thoracic bioimpedance	Respiration	Research
BX100	Koninklijke Philips N.V., Amsterdam, the Netherlands	Patch, thoracic bioimpedance	Respiration	Medical
µCor3	ZOLL Medical Corporation, Chelmsford, MA, USA	Patch, thoracic RF^c^ impedance 0.5-2.5 GHz	Heart failure	Medical
Physioflow	Manatec Biomedical, Poissy, France	Chest module, thoracic bioimpedance	ICG^d^	Medical

^a^BIVA: bioelectrical impedance vector analysis.

^b^BIA: bioelectrical impedance analysis.

^c^RF: radiofrequency.

^d^ICG: impedance cardiography.

## Basic Principle of Bioimpedance

### Overview

Our aim is to address the clinical application areas for wearable bioimpedance. The aim is not to discuss the technology in full detail; a comprehensive description on bioimpedance is provided elsewhere [24,25]. However, to understand the opportunities and challenges for clinical applications, some background on the technology is needed. Therefore, we first provide a brief overview of the principles of bioimpedance measurements.

Bioimpedance is a method to assess the electrical properties of a tissue. Different tissues such as the bone and fat have different electric properties. In 1996, more than a century after the initial work on electrical properties of biological tissues in 1872 [26], Gabriel et al [27] reported the measurement of dielectric properties of different biological tissues over a large frequency range (10-20 GHz). These experiments and observations formed the basis of subsequent bioimpedance research in various applications.

Bioimpedance reflects the extent to which the living tissue impedes the flow of electrical current. The electrical properties of biological tissue are determined by the characteristics of the extracellular fluid (ECF), cell membranes, and intracellular fluid (ICF). To study the electrical properties, an alternating current with a single frequency measurement or range of frequencies is injected into the tissue and the opposition of the tissue to this current flow (ie, the bioimpedance) is measured.

**Figure 1:**
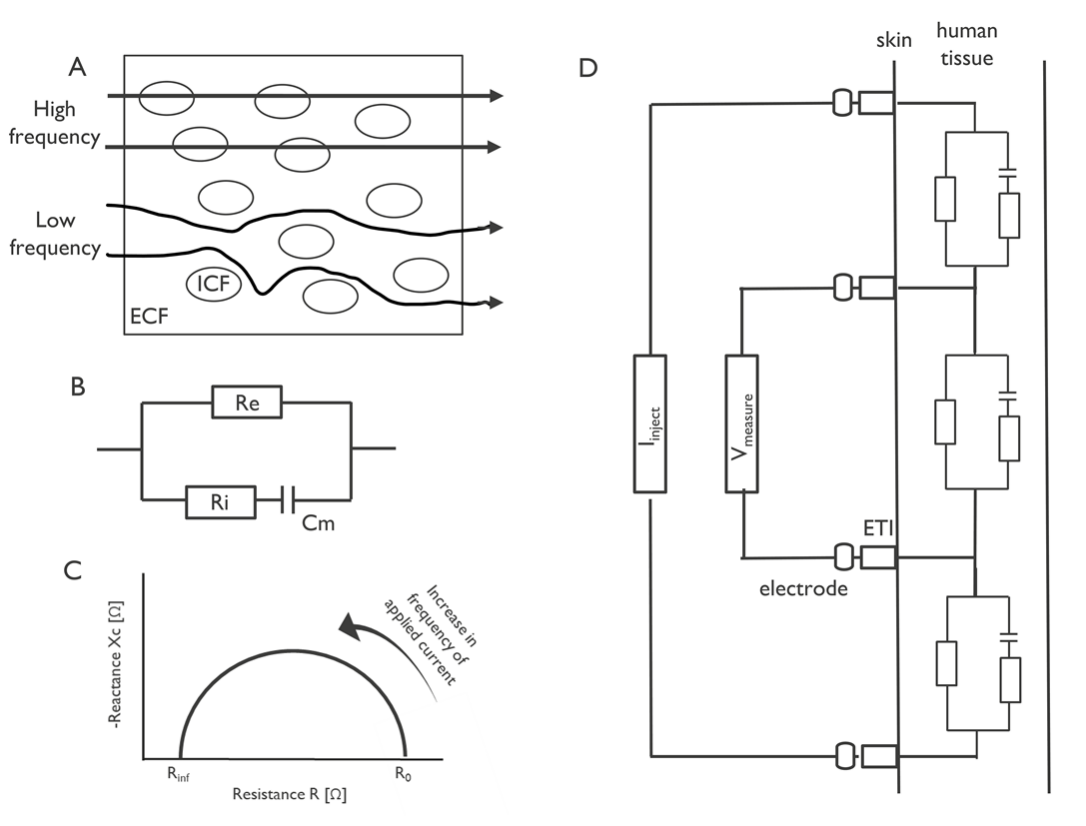
(A) Low-frequency current travels around the cell, while high frequency current can penetrate cells. (B) Electrical model of the tissue with extracellular resistance (Re), intracellular resistance (Ri), and conductance representing the cell membrane (Cm). (C) Illustration of bioimpedance spectroscopy data visualized in the R-Xc plane. Increasing frequencies of the injected alternating current appear counterclockwise in the plot. (D) Tetrapolar electrode configuration in bioimpedance measurement. ECF: extracellular fluid; ETI: electrode tissue impedance; ICF: intracellular fluid; Iinject: injected current; Vmeasred: measured voltage.

Bioimpedance measurements at any frequency are expressed as a complex number, with the real part referred to as the resistance and the imaginary part referred to as the reactance. The resistance is regarded as a measure of the obstruction to an electrical current, whereas the reactance is related to the storage of the electrical current. The resistance is attributed to the fluids in the tissues (including the therein dissolved ions) and the capacitance is attributed to the cell membrane. Since the resistance of the cell membrane is very small, it is often neglected (see electrical scheme in Figure 1B). At low frequencies of the injected current, the current does not penetrate the cells, but mainly flows through the ECF; thus, bioimpedance measurements at low frequency can be used to gain insight into the ECF. However, when using high frequencies for the injected current, the current flows through the cells, and thus the measurements provide insights into both the cellular and the extracellular components (Figure 1A).

The resistance and reactance can be used to calculate the phase angle and the magnitude. The phase angle is calculated by the arc tangent of the ratio of reactance and resistance at a certain frequency. The phase angle is therefore considered to be a useful metric for cellular health, and is expected to be an indicator of the cellular integrity, cell mass, and extracellular versus intracellular water content. The magnitude is calculated as the square root of the sum of the two vectors.

In general, bioimpedance has been applied to three types of problems: (1) dynamic monitoring, applied mainly in the chest to monitor respiration and hemodynamic parameters; (2) slowly evolving parameters such as body composition monitoring; and (3) electrical impedance tomography (EIT) or imaging. To address these application areas, different types of bioimpedance measurements have been developed. There are several measurement methods with various numbers of electrodes, namely 2, 3, and 4. Here, we only describe the tetrapolar configuration with 4 electrodes since this minimizes the effect of electrode tissue impedance (ETI), which is undesired in real-life bioimpedance measurements. Measurements using a tetrapolar electrode configuration and a single frequency of the injected current are applied to assess either dynamic changes in vital parameters or body composition. The latter measurements are referred to as single-frequency bioimpedance analysis (SF-BIA). A second approach to assess body composition is through multifrequency bioimpedance measurements, either through multifrequency bioimpedance analysis (MF-BIA) or bioimpedance spectroscopy (BIS) [26,28]. Finally, EIT measurements are performed using either single or multiple frequencies of the injected current and an array of at least 8 electrodes.

### Single-Frequency Measurements for Dynamic Monitoring

To obtain the bioimpedance of a tissue, an alternating current is applied to the tissue. Electrodes are placed on the surface of the skin to ensure electrical contact with the tissue. As mentioned above, a tetrapolar electrode configuration is often used to circumvent the effect of ETI. In such a configuration, two electrodes are dedicated for the current injection and the other two are used for obtaining the voltage measurement (Figure 1D). The configuration, or positioning, of the electrodes together with the electrical properties of the underlying tissue will determine the current path of the injected current through the body. For example, current injected through electrodes positioned on the thorax will flow through part of the thorax underlying these electrodes. Therefore, electrode positioning is an important step in the design of the bioimpedance measurement.

**Figure 2:**
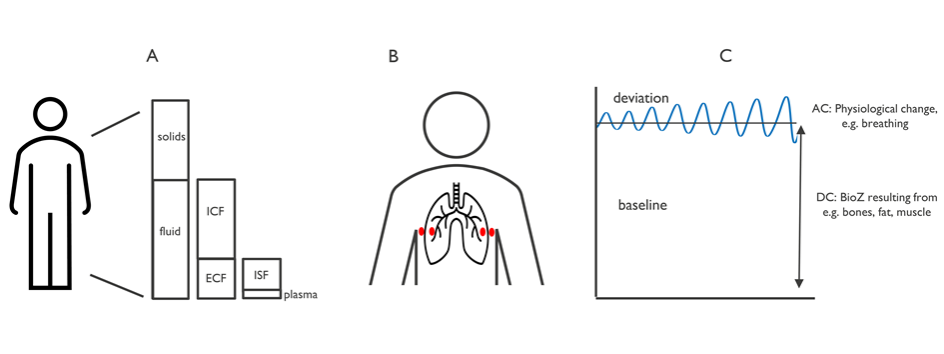
(A) Illustration of body composition consisting of solids (eg, bone, dry cell mass) and fluids. The fluids consist of intracellular fluid (ICF) and extracellular fluid (ECF), with the latter comprising interstitial fluid (ISF) and plasma. (B) Electrode configuration example of respiration monitoring with the measured bioimpedance (bioZ) signal. (C) The measured bioZ signal contains a dynamic component (AC) resulting from physiological changes such as breathing and a baseline component (DC) resulting from tissues (eg, bones, fat and/or muscle).

Longitudinal thoracic bioimpedance measurements can be performed to assess respiration or hemodynamic parameters. The measured thoracic bioimpedance signal contains a baseline component and a dynamic component. The baseline component is a constant bioimpedance value that is determined by the tissues (eg, the adipose tissue) and does not change during the measurement over several minutes. The dynamic component is related to dynamic changes in the tissue during the measurement (Figure 2). During a measurement of several minutes, a subject breathes and the heart pumps blood through the thorax. Airflow moving in and out of the body and pulsatile blood flow modulate the electrical properties and thus the measured bioimpedance signal. The electrode configuration, by affecting the measured tissue volume, and the frequency of the injected current, by affecting the current path, both influence the baseline and dynamic components of the measurements.

### Body Composition Monitoring

The previous section described monitoring of dynamic changes, or the dynamic component of the signal, whereas body composition monitoring is related to the baseline component of the measurement. Body composition parameters obtained through bioimpedance measurements include fat percentage and total body water (TBW) content or the hydration status. TBW is the sum of the extracellular water (ECW) and intracellular water (ICW) content. Reference methods for estimating TBW, ECW, and ICW, such as dilution of radioactive deuterium, bromide, and radioactive potassium, are invasive and expensive. These methods also must be applied under clinical supervision and are not suitable for frequent or ambulatory monitoring.

The simplest method for bioimpedance body composition monitoring is SF-BIA, which is used to estimate TBW, ECW, ICW, and fat free mass (FFM) using statistical analysis. The frequency of the current is set to 50 kHz. SF-BIA is applicable for normal hydrated subjects [29], which uses the inversely proportional relationship between assessed bioimpedance and TBW. SF-BIA first predicts the TBW and FFM using two statistically derived equations [29], and then estimates the ECW and ICW to be 75% and 25% of the TBW, respectively. To improve the body composition estimation, bioimpedance vector analysis (BIVA) was introduced, which also uses single-frequency bioimpedance measurement, mainly at 50 kHz, but the data are normalized to the length of the subject. BIVA provides information about changes in both tissue hydration and soft-tissue mass. However, similar to SF-BIA, BIVA does not provide any quantitative estimate of tissue mass (in kilograms) or fluid volumes (in liters). Therefore, MF-BIA was developed to exploit the frequency dependence of the different tissues. MF-BIA uses a similar approach to SF-BIA, except that it applies a spectrum of frequencies to the body tissue and performs multivariate statistical analysis to estimate TBW, ECW, ICW, and FFM. In contrast, BIS predicts ECW and TBW by determining the resistance at zero frequency (R_0_) and infinity frequency (R_inf_). BIS provides quantitative results on TBW, as well as on ECW and ICW. Typically, a larger number of different frequencies is used in BIS measurements compared to MF-BIA. The measured response at these frequencies is displayed in the R-Xc plane (plotting resistance vs reactance), as shown in Figure 1C.

Several empirical electrical models have been developed to analyze these measurements. Over the years, different variations of these empirical models have been presented [30,31]. Although these models can describe the data, they are not a true representation of the underlying physiology. One of the earliest models is the Fricke and Morse model [32], which consists of two resistors, ECF resistance and ICF resistance, and a resistor in parallel with a capacitor, which represents the cell membrane. This model has a direct physical interpretation. The most widely used model is the Cole-Cole model [33]. To account for the nonideal capacitive behavior of cell membranes, an additional parameter (α) was added to this model. Although this improved the accuracy of the fit, the interpretability of the model was reduced. The resistance values at infinitely low R_o_ and high R_inf_ are easily derived from the analysis and relate to ECF and TBW.

### EIT Measurements

EIT originates from the 1970s [34] and is an imaging technique with relatively low resolution when compared with traditional imaging techniques such as magnetic resonance imaging (MRI) or computed tomography (CT). However, EIT has the advantage of low costs, low power, no radiation, a high temporal solution, and the potential to be wearable [35].

EIT estimates the conductivity distribution within a given volume. The measurement exploits the fact that different tissues vary in their electrical properties. To assess the conductivity distribution, EIT uses electrical alternating currents injected from the surface area of the volume. Toward this end, electrodes are placed around the surface of the volume of interest (eg, the thorax). Currently, EIT systems often consist of 8 to 16 electrodes per ring of electrodes. The electrodes used for current injection and voltage measuring are continuously changed in specific patterns. The measured voltages are used in the reconstruction of the image, which is an ill-posed nonlinear inverse problem. Two types of images can be derived from EIT measurements: a difference or an absolute image. Difference images are created by measuring the same volume multiple times and then subtracting and dividing by a reference dataset. The reference dataset can be generated with the same measurement setting, but the data are collected at a different moment in time (time-difference EIT) or with a different frequency of the injected current (frequency-difference EIT). The reconstruction will lead to the difference image, which may be relevant during respiration monitoring. The absolute image shows the absolute properties of the area of interest. Several groups have developed solutions for image reconstruction, including the freely available software EIDORS [36,37]. Initially, image reconstruction was performed in a 2D manner using a single ring of electrodes. Subsequent methods have been developed for 2.5D or 3D reconstruction using multiple rings of electrodes covering a volume [38].

## Application Areas of Bioimpedance Monitoring for Chronic Conditions

### Applications of Focus

Owing to its versatile nature, wearable bioimpedance can be used for a wide range of clinical and lifestyle applications, which include body composition monitoring, monitoring of hemodynamic parameters, respiratory monitoring, and imaging. Here, we focus on the use of wearable bioimpedance monitoring in chronic diseases. This section is divided in three parts: monitoring dynamic parameters, slowly evolving parameters, and imaging.

### Dynamic Parameters in the Chest

### Overview

Dynamic changes in thoracic impedance consist of two parts: a respiratory and a hemodynamic or cardiac contribution. Impedance pneumography monitors the changes induced by respiration in the impedance of the thorax, whereas impedance cardiography measures the changes due to the cardiac contribution. In measuring either component, the other is typically regarded as a disturbance of the signal.

### Impedance Pneumography

Currently, respiratory status is assessed in clinical practice in patients with chronic obstructive pulmonary disease (COPD), asthma, and sleep apnea. In patients with COPD and asthma, a spirometer is used to assess respiratory function. Spirometer tests require a face mask or mouthpiece and trained medical personnel to perform the test well. These prerequisites make the test obtrusive and unsuitable for ambulatory monitoring. Similarly, sleep apnea is diagnosed in a sleep lab using polysomnography with many cables, requiring a complex set up. For these reasons, less invasive methods are being investigated that can provide continuous and ambulatory monitoring in a comfortable and unobtrusive manner. Impedance pneumography is being studied as one such a technology.

**Figure 3:**
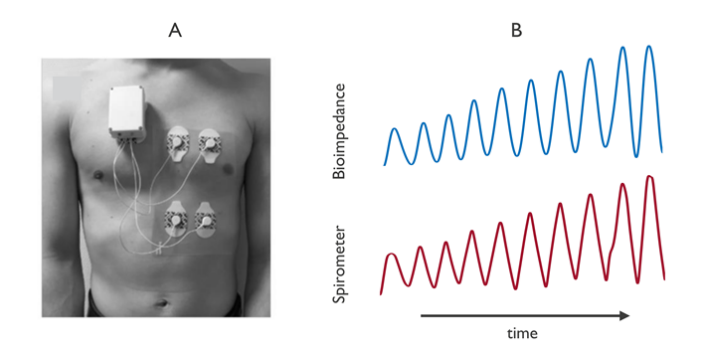
Example of a wearable device (imec the Netherlands, Eindhoven, the Netherlands). (B) Example impedance pneumography data. The figure shows the similarity between bioimpedance and spirometer data for an increasing respiratory volume protocol.

During the impedance pneumography measurement, electrodes are placed on the chest to obtain the thoracic bioimpedance (Figure 3). These electrodes can be attached with lead wires to a device or integrated in a patch. The dynamic component of the measured signal relates to the varying electrical properties in the chest, encompassing breathing. In the measurement, an aggregate signal is measured of the underlying tissue. This volume comprises not only the lungs but also other tissues of the thorax, such as the muscles and the fat. To determine the applicability of the signal, it is necessary to understand the different contributions to, or the origin of, the signal. Some studies have investigated the contributions of the underlying tissue to the measured bioimpedance signal using either animal models or computer simulations [39-43]. Animal studies from the 1960s and 1970s focused on the contributions of chest movement and respiratory volume to the bioimpedance signal [42,43]. Subsequent studies in human subjects showed that during normal breathing, the relation between volume and bioimpedance appeared to be linear [44-48]. However, during abnormal breathing, the relation between volume and bioimpedance appeared nonlinear, indicating the contribution of additional components to the signal. These contributions can be seen during sleep apnea events monitored with bioimpedance [49]. Recently, Blanco-Almazan et al [50] showed that both respiratory volume and chest movement contribute to the bioimpedance signal during normal breathing and during inspiratory loading conditions, with the contribution of chest movement becoming more important when muscle activity was the highest.

Another topic of research has been the effect of electrode positioning, which has been studied using computational modeling and data collection in volunteers. The electrode positioning influences the volume that is being investigated. Finite element models of (part of) the thorax have been constructed, in which the electrode position was varied to optimize the measurement location for monitoring the lung area [39-41]. Using this approach, positions were compared in terms of sensitivity (percentage contribution of the lung tissue to the measured bioimpedance signal) and specificity (the amplitude of the lung contribution). The simulations showed that the electrode locations around the middle of the thorax reflect impedance changes in the lung region. Data were collected on human volunteers using different electrode configurations and were compared against those obtained using a reference device (eg, a spirometer) [45,46,48,51]. This comparison also showed that the locations around the middle of the thorax were able to accurately capture respiration.

Several studies have assessed the applicability of impedance pneumography for respiration monitoring in chronic conditions. In children, nocturnal impedance pneumography measurements can be used to monitor the increased tidal flow variability as associated with childhood asthma risk [52]. In addition, it was shown that impedance pneumography and direct pneumotachograph measurements had a similar relation with lung function in infants with respiratory symptoms. However, in infants with clinically observed airway obstruction, the measured tidal breathing flow parameters differed between impedance pneumography and direct pneumotachograph [15], which further support that factors other than volume contribute to the bioimpedance measurement.

In adults, impedance pneumography has been applied in patients with COPD and sleep apnea. In patients with COPD, bioimpedance measurements were combined with electromyography and mechanomyography measurements assessing muscle activity [53], showing the applicability of noninvasive multimodal respiratory assessment. Regarding sleep apnea, recent work evaluated a shirt with ECG and bioimpedance for monitoring in healthy volunteers [54]. In addition, in patients with sleep apnea, a wearable bioimpedance device was able to detect apnea events, which opens opportunities for unobtrusive screening, diagnostics, and treatment monitoring in sleep apnea [49]. Finally, in the hospital setting, impedance pneumography is currently available for respiratory monitoring, although typically in a nonwearable form. An example is the ExSpiron Minute Ventilation System (Respiratory Motion Inc), which has been tested in the postanesthesia care unit and the intensive care unit [55,56].

### Impedance Cardiography

**Figure 4:**
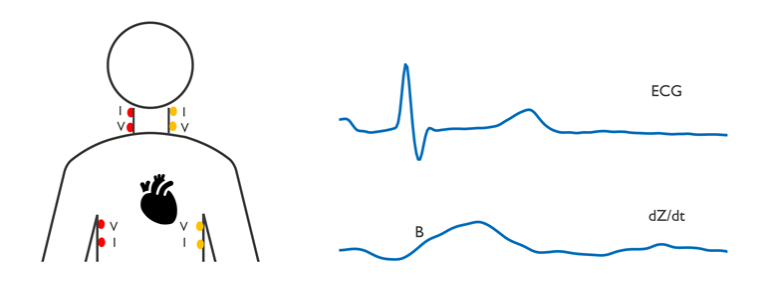
Left: Electrode configurations for impedance cardiography (ICG) measurement, using either red electrodes or yellow electrodes, with current injection electrodes (I) and voltage electrodes (V). Right: electrocardiogram (ECG) and ICG signals showing characteristic morphology with the B point as an example.

Cardiac output is related to how much blood the heart delivers to the body, which is measured to assess the status of the heart, relevant in many chronic conditions such as heart failure. Cardiac output can be assessed with several technologies such as Doppler echocardiography and intracardiac catherization. Echocardiography is time-consuming and requires trained medical personnel, whereas catherization is invasive. Impedance cardiography has been proposed as a noninvasive and potentially ambulatory method to assess hemodynamic parameters such as cardiac output. The possibility to measure hemodynamic parameters noninvasively with impedance has been studied for a long time [14]. In general, 4 electrodes are used for the impedance measurement to assess changes in thoracic bioimpedance related to the cardiac cycle. The ECG signal is collected simultaneously to time the cardiac events. Different electrode configurations have been proposed to measure the impedance cardiography signal. Initially, four band electrodes were used, with two electrodes positioned around the neck and two around the abdomen. These band electrodes were subsequently replaced by round electrodes [57] (see Figure 4). Alternative electrode configurations have been evaluated. For example, one configuration positions one electrode on the forehead, the lowest one above the leading edge of the heart, and the remaining two in between [58]. Desktop devices are typically used for these measurements, but some studies have also investigated wearable devices [59-62].

The measured impedance signal (*Z*) varies with the contraction of the heart. In the derivative of the signal (*dZ/dt*), different points have been shown to correspond with different parts of the cardiac cycle, such as the B-point with the opening of the aortic valve. The measured impedance and its derivative are used in formulas for an approximation of stroke volume. The first such model was presented by Kubicek, which was modified in subsequent studies [63]. All models to derive stroke volume use assumptions such as those related to the shape of the thorax (cylinder or truncated cone), current path, blood resistance, and origin of the pulsatile impedance changes. To fully understand the signal and its applicability for clinical monitoring, the origin of the signal needs to first be understood; however, the origin of this signal seems to be complicated and has led to controversy in the field. Recently, de Sitter et al [64] compared different mathematical models that aim to understand the underlying physiological signals that contribute to the change in bioimpedance used in impedance cardiography in a systematic review. This comparison showed no consensus in the origin of the change in the bioimpedance signal, highlighting the complexity and the controversy around this topic.

At the same time, many studies have tried to validate this technique on different clinical use cases. A portion of these studies showed good results in the comparison of impedance cardiography with standard clinical methods such as the invasive thermodilution pulmonary artery catheter [65-67], whereas other studies showed insufficient agreement between the measurements [68-70]. In addition to validation studies, the potential role of impedance cardiography in disease diagnosis and disease management has been evaluated. For disease management, not only absolute values are of interest but also relative changes in stroke volume or cardiac output. For example, in stable heart failure patients, regular impedance cardiography measurements have been shown to have predictive value for near-term recurrent decompensation [71].

Stroke volume and cardiac output monitoring are of substantial interest for many diseases. The use of impedance cardiography to assess these parameters has gained interest because of its advantages of noninvasiveness, relatively low cost, and relative simplicity. However, there is still no consensus on the origin of the signal. In addition, the results for absolute monitoring are inconsistent. Many studies have assessed validation in different use cases and the applicability for different diseases, with mixed results. Therefore, further work is needed to fully understand the signal and its applicability for chronic disease.

### Slowly Evolving Parameters

Besides monitoring dynamic parameters, bioimpedance is used for monitoring more slowly evolving parameters such as body composition and fluid status. Measurements related to body composition are reflected by the baseline component of the bioimpedance measurement. These measurements are often performed with benchtop devices measuring total body impedance from the hand to foot, using models (eg, the Cole-Cole model as explained in the Principle of Bioimpedance section above) to convert the impedance values into body composition parameters. In the domain of chronic diseases, these measurements have been used to assess malnutrition or body fluids, such as overhydration, dehydration, or local fluid buildup (eg, pulmonary edema). Early attempts to evaluate pulmonary edema in patients with bioimpedance originate from the 1970s [72]. More recently, wearable bioimpedance has been used for fluid monitoring such as in patients with congestive heart failure and those with ESKD undergoing hemodialysis treatment [16,73].

Hemodialysis is a life-saving treatment for patients with ESKD. However, in these patients, mortality levels are high and many patients suffer from cardiovascular complications. The nature of hemodialysis treatment (three 4-hour treatments per week) results in large fluid changes in the patient. Fluid builds up during the interdialytic period and is rapidly extracted during the 4-hour hemodialysis treatment. In patients with ESKD, fluid overload in the interdialytic period is associated with a higher cardiovascular risk, disease progression, and a rise in cardiovascular morbidity and overall mortality [74-76]. Maintaining optimal fluid balance in the body of a patient with ESKD is still a challenge. In current clinical practice, treatment is based on the dry weight of a patient, but objective dry weight assessment is currently lacking in clinical routine practice. Bioimpedance monitoring could play a role in maintaining fluid balance in patients with ESKD and has been associated with improvement of cardiovascular parameters [12,77,78] in studies using benchtop devices. Being able to unobtrusively and continuously monitor the fluid status could provide even larger value. Studies have shown that local wearable thoracic bioimpedance measurements can be used to accurately track fluid and weight loss during hemodialysis [16,79], but future work is needed, including exploring the potential for monitoring at home.

At the same time, studies have focused on wearable bioimpedance fluid monitoring in patients with congestive heart failure. In these patients, the pumping capability of the heart is reduced, and fluid can build up in the lungs or the extremities. Bioimpedance monitoring has also been used to assess pulmonary congestion. Several studies have shown the benefit of daily and continuous monitoring in patients with congestive heart failure in the form of portable benchtop devices [80,81], wearables [73,82-84], or even implantables [85,86]. Wearable bioimpedance monitoring predicted decompensation and hospitalizations [8,87]. Moreover, wearable bioimpedance monitoring was shown to be a useful marker for 30-day mortality and rehospitalization after diuretic treatment during hospitalization in patients with congestive heart failure treated with diuretic therapy [84].

Bioimpedance measurements, in combination with empirical models, have also been frequently used to study body composition in terms of muscle mass and body fat content, and are available in clinical settings through devices such as Maltron BodyScan 920-II (Maltron International Ltd) and Fresenius Body Composition Monitor (Fresenius Medical Care Pte Ltd). These measurements have also been used to assess malnutrition [88]. However, since slowly evolving processes underly these applications, they have mainly been studied with benchtop devices.

### Imaging Using EIT

Medical imaging enables gaining a view of the inside of the body. There are many imaging modalities currently available, such as MRI, radiography, ultrasound, and functional near-infrared spectroscopy. Some of these modalities use radiation (such as radiography), expensive equipment (such as MRI), or trained personnel. Therefore, some of these techniques only allow for obtaining a snapshot of the status of the patient. Imaging using EIT has the advantage of being continuous, low cost, low power, and with wearable potential, but the spatial resolution of the image of this modality is relatively low in comparison with that of other imaging modalities such as CT and MRI. Currently, the main use of EIT is during mechanical ventilation, which is used to monitor the ventilation of both lungs to protect the patient from lung damage caused by the ventilator. With respect to chronic conditions, the use of EIT in other application areas shows potential but requires more research. In patients with pulmonary conditions such as COPD, EIT could provide the spatial distribution of the pulmonary function to enable tracking regional lung function over time or after an intervention such as respiratory muscle training or the use of a bronchodilator [89]. It was suggested that monitoring spatial differences could improve patient phenotyping, monitoring disease and treatment effects, and predicting clinical outcomes [37,89]. EIT could also bring value to other chronic diseases such as epileptic monitoring and stroke. Being able to continuously monitor brain activity could help in locating the regions of the brain involved in epileptic seizures [90]. Hand gesture recognition could aid in physical disabilities such as those present after stroke [13].

One of the potential benefits of EIT is that it could be wearable. Currently, there are no wearable EIT systems on the market, but several research prototypes have been developed [13,91-94]. These wearable systems have been shown to capture respiration [92,93]. Another system combines EIT with multilead ECG in a vest, thereby opening up the possibility to measure respiration and impedance changes from the cardiac region [91]. In a very different application area, Zhang et al [13] used EIT for hand gesture monitoring.

To date, besides during mechanical ventilation, EIT has mainly been applied in controlled laboratory settings. Before wearable EIT can be applied for the monitoring of chronic conditions, further steps in development and validation need to be taken, such as in handling the imaging during daily living conditions. Finally, the added clinical value needs to be demonstrated for the different application areas before the technology will be widely adopted.

## Challenges and Outlook

### Hurdles for Broad Application

Electrical principles have long been used in the medical field, and bioimpedance measurements have been explored for many decades. Initially, these measurements were performed with large benchtop devices, but more recently have been assessed in wearable form with consideration of many different applications, ranging from respiratory volume to cardiac output and from body composition monitoring to imaging. The relatively low cost of the system and its noninvasiveness make bioimpedance an interesting sensor technology for wearable monitoring of chronic conditions. However, there are still several hurdles to be overcome before bioimpedance will be widely adopted in clinical practice. Some of these hurdles apply to wearable bioimpedance, while others do not solely apply to bioimpedance monitoring but should be considered in the broader context of all wearable monitoring techniques. Here, we will discuss both aspects.

### Challenges and Limitations of Wearable Bioimpedance

As indicated in the preceding sections, wearable bioimpedance is a promising technique owing to its advantages of low cost, wearability potential, and noninvasiveness. However, like all technologies, wearable bioimpedance has some disadvantages. First, bioimpedance measurements, as is the case for many wearable measurements, are prone to motion artifacts. Collecting data in a real-world environment is prone to activities leading to these artifacts. Corrupted data can lead to misinterpretation of the state of a patient; therefore, solutions to either prevent or remove these artifacts are crucial. To prevent motion artifacts, one might ask the subject to sit still during a measurement to capture slowly evolving parameters, which may be performed once a day for approximately 1 minute, such as for measuring body composition. Alternatively, measurements could be triggered or filtered at certain postures or activity levels [95,96]. However, this is not suitable for ambulatory monitoring of dynamic parameters such as respiration monitoring, when one is interested in respiration throughout the day. The two main strategies to handle corrupted data in impedance pneumography are so-called “quality indicators” that exclude corrupted data or to try to salvage corrupted data by using motion artifact reduction techniques. Motion artifact reduction is often applied to ECG and photoplethysmography signals [97-100], but to a lesser extend to impedance pneumography signals. For example, Ansari et al [101] compared different methods for different types of movement. Regarding the so-called quality indicators, Charlton et al [102] reviewed the methods of obtaining quality indicators for respiratory signals and concluded that further research is needed to design powerful quality indicator algorithms for different applications [102]. Recently, they also published a quality assessment method for impedance pneumography signals [103]. Similar to impedance pneumography, artifact handling is relevant for impedance cardiography measurements, including both absolute and relative measures. Artifacts in the impedance cardiography signal make it more difficult to detect the fiducials such as the B-point in the signal, resulting in less accurate estimates of stroke volume and cardiac output. Three approaches for artifact handling have been used in the analysis of impedance cardiography signals: artifact detection [104], artifact reduction [105-107], and posthoc outlier removal from estimated parameters [108].

As mentioned above, the electrode positioning influences the measured volume. As such, changing the electrode position slightly will lead to a change in the measured volume and thus changes the measured impedance value. This is relevant when looking at small changes over measurements that require exact electrode repositioning, but is not important when looking at derived metrics that are not related to the signal amplitude such as the respiratory rate. To circumvent the effect of electrode positioning on the absolute measured value, algorithmic solutions should be developed to correct for these differences or electrode position, and independent metrics should be developed.

In addition, measurements that do not require device or electrode reattachment are subject to change. The condition of the skin can change over time as can the adhesive capability of the electrodes. Adhesive materials for electrodes are optimized for their maximal comfort and endurance.

Finally, bioimpedance is affected by body composition. Depending on the body composition, the current path through the body will differ. In the case of obesity, the current would need to penetrate a larger layer of fat before reaching the underlying tissues, which will affect the measured impedance values. Since there are many different body shapes, personalization of the measurements could circumvent this issue.

### General Challenges for Wearables and Wearable Bioimpedance-Based Devices

Although there are some wearable devices on the market for clinical use, such as Holter devices and cardiac rhythm monitoring patches, most wearables have not yet been approved for medical use. Their use has mainly been studied in the research domain thus far [17]. Tests in a controlled or laboratory environment may not represent use in the real world, and validation in resting conditions may not represent (daily life) motion situations [18]. Therefore, there is a need to collect real-word evidence. There is also variability between devices [17], indicating the need for standardization of evaluation of wearables in the assessment of reliability, sensitivity, and validity of the data [18], further signaling the necessity to collect real-word evidence.

Dinh-Le et al [22] reviewed the integration of wearable technology from electronic health records. One associated challenge with this approach is related to the large streams of data that must comply with all privacy and security standards. In addition, patients fear misuse of their data, potentially leading to discrimination and changes in coverage by insurance companies. Therefore, patients should be well informed on the data collection and handling procedures. In addition, proprietary and closed systems pose difficulties with regard to system interoperability and connectivity.

The data streams generated by wearable devices that are often worn 24/7 present another challenge. The current health systems are not prepared for handling such high volumes of rapidly accumulating data [109]. Besides data storage, the vast amount of data is another major challenge, as the data also need to be analyzed. Clinical decision support systems have been implemented that generate false alarms in some cases such as drug interactions or elevated blood pressure. Reliability of these alarms is crucial, since health care providers could experience alarm fatigue due to the large number of false alarms [110,111]. AI is believed to have great potential in the field of clinical data analytics [112]. Regarding bioimpedance monitoring, AI has been shown to be able to detect sleep apnea events [49] and to estimate dry weight in pediatric patients on chronic hemodialysis [113]. However, these algorithms should not stop at classification but should further lead to actionable insights for the health care provider or the patient. Integration of these algorithms on the devices could help in achieving the ultimate goal of developing closed-loop care-providing wearables [112].

As indicated above, the use of many wearables for clinical applications is currently limited to the research domain. It is widely acknowledged that for further acceptance and integration into clinical practice, the proof of medical benefit of wearables through dedicated medical trials is needed [9,18,22,23,112]. Additionally, health care cost should be evaluated for the long and the short term. Wearables are often proposed as a solution against rising health care costs [9,114]. However, there are also examples that show an increase of health care resource utilization with wearables [20].

Interestingly, the view of patients has been less well studied from these aspects. Tran et al [19] explored the perspectives of patients on wearable devices and AI in health care in France. Their study showed that half of the patients felt that digital technology and AI techniques are an important opportunity. However, the study also showed that the patients are not ready for fully automated care. One out of three patients refused one of the devices or AI systems, and patients highlighted the risks regarding privacy and data misusage, the absence of a human interaction and relations, and uncertainty of reliability.

The role of the patient has also been highlighted in studies using technology showing low compliance and large dropouts [115,116]. Nevertheless, other studies have shown good compliance [117]. Interestingly, studies have indicated that patients can experience monitoring as obtrusive and undesired, and that it can even lead to higher depression scores [118]. Several studies have focused on increasing the comfort level and decreasing the obtrusiveness of wearables to circumvent these problems, looking at the possibility to integrate bioimpedance measurements in clothing [54], the use of flexible and stretchable materials [119], and to increase battery life [82]. In some use cases, “nearables” could be used as an alternative to wearables, leading to invisible and effortless methods. One such example is the integration of bioimpedance measurements in chairs or beds via capacitively coupled bioimpedance [120].

## Summary and Prospects

Although bioimpedance monitoring is not a new concept, wearable bioimpedance monitoring for chronic conditions is a relatively new field. In this viewpoint, we have shown the potential of bioimpedance monitoring in application areas such as respiration, cardiac, body composition, and fluid monitoring, as well as the remaining challenges that need to be addressed before it can be widely adopted in the medical field. Nevertheless, wearable bioimpedance monitoring has large potential to change monitoring and disease management for patients suffering from chronic diseases such as respiratory, cardiac, or kidney disease by enabling low-cost and low-power home-monitoring solutions. These developments can further have an impact on health care costs and quality of life of patients with chronic diseases.

## Abbreviations

AI: artificial intelligence
BIS: bioimpedance spectroscopy
BIVA: bioelectrical impedance vector analysis
COPD: chronic obstructive pulmonary disease
CT: computed tomography
ECF: extracellular fluid
ECG: electrocardiogram
ECW: extracellular water
EIT: electrical impedance tomography
ESKD: end-stage kidney disease
ETI: electrode tissue impedance
FFM: fat free mass
ICF: intracellular fluid
ICW: intracellular water
MF-BIA: multifrequency bioimpedance analysis
MRI: magnetic resonance imaging
NCD: noncommunicable disease
SF-BIA: single-frequency bioimpedance analysis
TBW: total body water
